# Risk of Stroke in Patients With Schizophrenia, Bipolar Disorder, and Major Depressive Disorder: A Cohort Study of 183,504 Subjects

**DOI:** 10.1111/acps.70043

**Published:** 2025-10-30

**Authors:** Mao‐Hsuan Huang, Chih‐Ming Cheng, Ju‐Wei Hsu, Ya‐Mei Bai, Tung‐Ping Su, Cheng‐Ta Li, Shih‐Jen Tsai, Yee‐Lam E. Chan, Mu‐Hong Chen

**Affiliations:** ^1^ Department of Psychiatry General Cheng Hsin Hospital Taipei Taiwan; ^2^ Division of Psychiatry Faculty of Medicine, National Yang Ming Chiao Tung University Taipei Taiwan; ^3^ Institute of Brain Science National Yang Ming Chiao Tung University Taipei Taiwan; ^4^ Department of Psychiatry Taipei Veterans General Hospital Taipei Taiwan

**Keywords:** bipolar disorder, cerebrovascular event, schizophrenia, severe mental illness, stroke

## Abstract

**Background:**

Major psychiatric disorder, including schizophrenia, bipolar disorder, and major depressive disorder, has been individually associated with increased risk of stroke. However, few studies have directly compared the stroke risk across these diagnostic groups within a unified cohort framework while accounting for stroke subtypes and relevant confounders.

**Methods:**

Using Taiwan's National Health Insurance Research Database, we identified 30,945 patients with schizophrenia, 30,360 with bipolar disorder, 30,447 with major depressive disorder, and 91,752 age‐matched controls without psychiatric illness between 2001 and 2009. Participants were followed until death or the end of 2011. Cox regression models were used to estimate the hazard ratio (HR) for ischemic and hemorrhagic stroke, adjusting for potential confounding factors. Sensitivity analyses were conducted by excluding stroke events occurring within the first 1 or 3 years of psychiatric diagnosis.

**Results:**

All three psychiatric groups exhibited significantly higher risks of ischemic and hemorrhagic stroke compared with controls. Stroke risk remained consistently elevated across age and sex strata for all psychiatric groups. Greater cumulative exposure to antidepressants was associated with reduced stroke risk across all three disorders; antipsychotics showed protective associations in schizophrenia and bipolar disorder, non‐lithium mood stabilizers were protective only in bipolar disorder, and lithium showed no significant association with stroke risk.

**Conclusion:**

Schizophrenia, bipolar disorder, and major depressive disorder are independently associated with increased stroke risk. These findings highlight the need for integrated vascular risk monitoring in psychiatric care.

**Summary**

- Significant outcomes
  ○ Schizophrenia, bipolar, and depression linked to increased stroke risk in a national cohort.
  ○ Stroke risk was elevated across all age and sex subgroups in psychiatric populations.
  ○ Higher cumulative exposure to antidepressants and antipsychotics was associated with reduced stroke risk.
- Limitations
  ○ Reliance on claims data may have limited adjustment for unmeasured confounders such as key lifestyle or clinical factors.
  ○ Pooling separately matched control groups into a single cohort may have introduced residual heterogeneity in between‐group comparisons.
  ○ Smoking status and stroke severity or outcomes were not fully captured, limiting risk estimation and prognostic interpretation.

## 1 Introduction

Stroke is a leading cause of mortality and long‐term disability, imposing substantial healthcare and societal burdens worldwide [1]. In addition to established risk factors such as hypertension, diabetes, and hyperlipidemia, emerging evidence has underscored the role of psychiatric disorders in the pathogenesis of cerebrovascular disease [2]. Individuals with severe mental illness, including schizophrenia, bipolar disorder, and major depressive disorder, face significantly elevated risks for stroke, as well as disparities in diagnosis, treatment, and secondary prevention [3, 4, 5].

Individually, each of these major psychiatric disorders has been associated with an increased risk of stroke. Meta‐analyses have reported a 1.55‐fold increased relative risk of stroke in patients with schizophrenia compared to non‐schizophrenia controls, potentially mediated by metabolic syndrome, unhealthy lifestyle behaviors, and the long‐term use of antipsychotic medications [6, 7]. Similarly, bipolar disorder has been linked to ischemic stroke, with proposed mechanisms including cardiometabolic comorbidities, systemic inflammation, and neuroendocrine dysregulation [8, 9]. Major depressive disorder has also been independently associated with both stroke incidence and stroke‐related mortality. A large meta‐analysis demonstrated that depression is associated with a significantly increased risk of both first‐ever and fatal stroke events [10, 11]. Biological explanations include dysregulation of the hypothalamic–pituitary–adrenal axis, endothelial dysfunction, and heightened platelet aggregation [12]. Behavioral mechanisms such as physical inactivity, poor diet, and reduced adherence to medical therapy may further exacerbate cerebrovascular vulnerability [13].

Although each psychiatric disorder has been individually linked to increased stroke risk, few studies have directly compared their relative risks within a single unified cohort. The lack of head‐to‐head investigations limits interpretability across diagnostic categories and obscures whether certain psychiatric populations face disproportionate vascular risk. Moreover, psychotropic medications represent a critical yet underexplored factor in the relationship between psychiatric disorders and cerebrovascular risk. Antipsychotics, particularly those associated with adverse metabolic profiles and cardiovascular effects, have been implicated in elevated risks of ischemic and hemorrhagic stroke [14, 15]. Mood stabilizers exhibit heterogeneous profiles, with evidence suggesting that lithium may confer neuroprotective or vascular‐protective properties, whereas valproate and carbamazepine have been linked to increased cerebrovascular events [16, 17]. Antidepressants, especially serotonergic agents, may influence platelet aggregation and bleeding risk, though findings remain inconsistent [18]. Given the widespread and long‐term use of these agents among patients with schizophrenia, bipolar disorder, and major depression, it is essential to account for their independent effects when estimating stroke risk.

Addressing this gap, we used Taiwan's National Health Insurance Research Database (NHIRD), a population‐based registry with near‐universal coverage, to examine stroke risk in an Asian cohort. Our design permits direct comparisons across schizophrenia, bipolar disorder, and major depressive disorder within a single matched framework, distinguishes ischemic from hemorrhagic subtypes, and incorporates psychotropic medication exposures, thereby providing a more comprehensive assessment of disorder‐specific and treatment‐related cerebrovascular risk.

### 1.1 Aims of the Study

Our study aimed to clarify the magnitude, subtype specificity, and medication‐related influences of cerebrovascular risk across major psychiatric disorders. We hypothesized that patients with schizophrenia, bipolar disorder, and major depressive disorder would each demonstrate elevated risks of stroke compared with non‐psychiatric controls.

## 2 Methods

### 2.1 Data Source

The present study utilized data from the NHIRD, a comprehensive nationwide repository established under the National Health Insurance (NHI) program, which has provided universal single‐payer healthcare coverage to nearly the entire Taiwanese population since 1995 (http://www.nhi.gov.tw/). By the end of 2010, the program encompassed approximately 99.6% of Taiwan's 23 million residents. The NHIRD, maintained by the National Health Research Institute, offers deidentified longitudinal health records, including demographic information, clinical diagnoses, and prescription data, collected using standardized coding systems such as the International Classification of Diseases, Ninth Revision, Clinical Modification (ICD‐9‐CM). Each insured individual is assigned an anonymous identifier, ensuring both patient privacy and the ability to track medical histories across time. This database has been widely utilized in numerous epidemiological studies in Taiwan [19]. For this study, a specialized psychiatric dataset within the NHIRD was used to identify individuals with severe mental disorders. The large, population‐based structure of the database reduces selection bias and enhances the generalizability of findings. Access to the NHIRD requires formal approval from regulatory bodies, and the current research protocol received ethical clearance from the Institutional Review Board of Taipei Veterans General Hospital, with informed consent waived due to the deidentified nature of the NHIRD (2018‐07‐016AC).

### 2.2 Study Population

**FIGURE 1:**
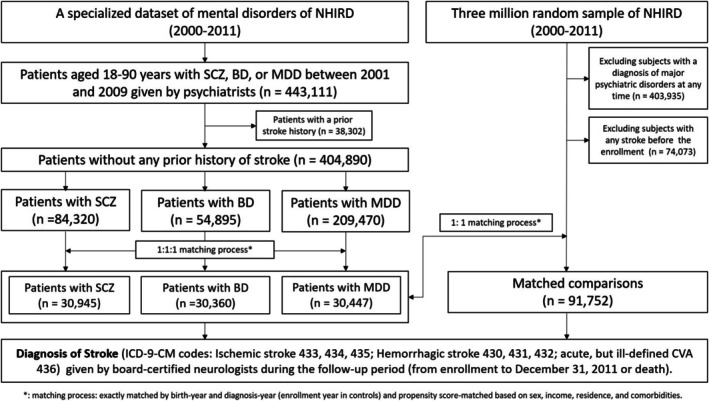
Study flowchart.

Adults aged between 18 and 90 years who were diagnosed as having schizophrenia (ICD‐9‐CM code: 295), major depression (ICD‐9‐CM codes: 296.2× and 296.3×) or bipolar disorder (ICD‐9‐CM codes: 296.0×, 296.1×, 296.4×, 296.5×, 296.6×, 296.7×, 296.80, 296.81, 296.89) by board‐certified psychiatrists at least twice between 1 January 2001 and 31 December 2009, and who had no history of stroke before enrollment, were identified from the specialized dataset of mental disorders in the NHIRD and were included in the psychiatric cohort. For patients with multiple psychiatric diagnoses, a hierarchical classification was applied with the following priority: schizophrenia > bipolar disorder > major depressive disorder. Patients were assigned to the highest‐priority diagnostic group to ensure mutually exclusive categories for analysis. The time of identification was defined as the diagnosis time of schizophrenia, bipolar disorder, or major depressive disorder. Exact matching (1:1:1) based on age (±180 days) and time of identification (±180 days) was performed between three groups (schizophrenia, bipolar disorder, major depressive disorder), and propensity score matching on demographics (except age) and medical comorbidities was used. A control cohort was then randomly chosen and exact matched to the psychiatric cohort in a 1:1 fashion in terms of age and identification time, excluding participants with previous diagnoses of major psychiatric disorders (ICD‐9‐CM codes: 295, 296, 297, 298, and 300) or any stroke (Figure 1).

### 2.3 Outcome Assessment

A diagnosis of stroke made by board‐certified neurologists was recorded during follow‐up (from enrollment until death or 31 December 2011, whichever came first). Stroke was defined either by ischemic stroke (ICD‐9‐CM code: 433, 434, 435) or hemorrhagic stroke (ICD‐9‐CM code:430, 431, 432). To enhance the diagnostic validity of stroke in this study, we adopted several established strategies. First, only individuals with a stroke diagnosis documented at least twice in outpatient visits or once during hospitalization were included, a method commonly used to reduce the likelihood of misclassification. Second, we limited stroke identification to cases in which the diagnosis appeared as the principal discharge diagnosis during inpatient admissions, thereby increasing diagnostic specificity. Lastly, the accuracy of stroke diagnoses in the Taiwan National Health Insurance Research Database (NHIRD) has been previously validated, with studies reporting a positive predictive value exceeding 90% for major cerebrovascular events when using similar case definitions [20].

### 2.4 Assessment of Covariates

Potential stroke‐related medical comorbidities were evaluated, encompassing alcohol‐related disorders (ICD‐9 codes: 291, 303, 305.0, 357.5, 425.5, 535.3, 571.0, or 571.1–571.3), hypertension (ICD‐9‐CM codes: 401–405), dyslipidemia (ICD‐9‐CM code: 272), diabetes mellitus (ICD‐9 code: 250), and traumatic brain injury (ICD‐9 codes: 800–804, 850–854, 959). Smoking was defined as having at least one prescription for nicotine replacement therapy (ATC N07BA01) or varenicline (ATC N07BA03) together with an ICD‐9‐CM diagnosis of tobacco use disorder (305.1) or history of tobacco use (V15.82). Each diagnosis required confirmation by corresponding physicians at least twice between 1 January 2001 and 31 December 2009 to ensure diagnostic accuracy. The Charlson Comorbidity Index (CCI) score, consisting of 22 physical conditions, was used to assess participants' overall systemic health and the severity of comorbid medical conditions at enrollment [21]. All‐cause clinical visits (the numbers of clinical visits per year) for the study groups and the matched‐controls cohort were included as a variable to account for potential detection bias. Income categories were defined as low (monthly income below New Taiwan Dollar [NTD] 15,840), medium (monthly income NTD 15,840–25,000), or high (monthly income > NTD 25,000), with NTD 15,840 representing tier 1 monthly salaries according to the 38‐tier insured amount designated by the NHI. Additionally, the level of urbanization (ranging from level 1 to level 5, with level 1 being the most urbanized and level 5 the least) was assessed [22]. The NHIRD records information on medication use according to the World Health Organization's Anatomical Therapeutic Chemical (ATC) classification, which allows for examination of dose–response associations between exposure to psychotropic agents and the risk of developing stroke. To facilitate comparisons across drugs, we adopted the defined daily dose (DDD) as a standardized unit of drug consumption. We further computed the cumulative defined daily dose (cDDD) for each participant over the follow‐up period. The DDD, as specified by the World Health Organization, reflects the assumed average daily maintenance dose for the principal indication of a given medication. For instance, the cDDD for lithium was derived by dividing the total amount of lithium prescribed during the study by its DDD value, where one DDD corresponds to 24 mmol of lithium. Each psychotropic drug's cDDD was subsequently stratified into three categories: < 30 cDDD, 30–364 cDDD, and ≥ 365 cDDD.

### 2.5 Statistical Analysis

For intergroup comparisons, Analysis of Variance (ANOVA) was utilized for continuous variables, and the Pearson *χ* ^2^ test was applied for nominal variables. The Levene test assessed variance homogeneity among groups. When variance distribution was uneven, Welch's one‐way ANOVA was used, followed by Games‐Howell post hoc pairwise comparisons. Three Cox regression models were used to investigate the hazard ratios (HRs) and 95% confidence intervals (CIs) for stroke occurrence between the study groups (schizophrenia, bipolar disorder, major depressive disorder) and the control groups: model 1 for the crude HR, model 2 for the adjusted HR after controlling for demographic data (age, sex, income, level of urbanization, all‐cause clinical visits), model 3 for the adjusted HR after controlling for demographic data, CCI score, smoking and medical comorbidities (traumatic brain injury, hypertension, dyslipidemia, diabetes mellitus, alcohol use disorder), and model 4 additionally adjusted for psychotropic medication use. Sensitivity analyses by excluding data from the initial 1‐year and 3‐year observations after the severe mental disorder diagnoses were conducted to validate our results. To explore whether hypertension and dyslipidemia mediated the association between major psychiatric disorders and stroke, we conducted causal mediation analyses using the CAUSALMED procedure. Separate models were specified for schizophrenia, bipolar disorder, and major depressive disorder, with ischemic and hemorrhagic stroke as outcomes, with the adjustment of all covariates. Hypertension and dyslipidemia were each modeled as binary mediators. The Cox proportional hazards model was applied to estimate natural direct effects, natural indirect effects, and natural total effects, with HRs and 95% CIs. The proportion mediated was calculated as the percentage of the total effect explained by the indirect pathway. A two‐tailed *p*‐value less than 0.05 was considered statistically significant. All data processing and statistical analyses were performed using the Statistical Package for Social Science (SPSS) Version 17 software (Chicago: SPSS Inc.) and Statistical Analysis System (SAS) Version 9.4 software (SAS Institute, Cary, NC).

## 3 Results

A total of 91,752 age‐matched individuals were included in the present study, comprising 30,945 patients with schizophrenia, 30,360 with bipolar disorder, 30,447 with major depressive disorder, and 91,752 controls (Table 1). The mean age at enrollment was approximately 41 years across all groups. The CCI score and frequency of all‐cause clinical visits were also elevated among psychiatric cohorts. Patients with schizophrenia, bipolar disorder, and major depression all exhibited a significantly higher incidence of ischemic stroke and hemorrhagic stroke than the control group. A higher proportion of patients with bipolar disorder received long‐term treatment with lithium and antiepileptic mood stabilizers, whereas patients with schizophrenia were more likely to receive long‐term antipsychotic treatment compared with the other groups.

**TABLE 1:** Demographic data and incidence of cerebrovascular accidents among patients and the control group.

	Control (*n* = 91,752)	Schizophrenia (*n* = 30,945)	Bipolar disorder (*n* = 30,360)	Major depression (*n* = 30,447)	*p*	Post hoc
Age at enrollment (years, SD)	40.6 (14.3)	40.5 (14.3)	40.6 (14.3)	40.8 (14.3)	0.103	
Sex (female, %)	52,459 (57.2)	17,142 (55.4)	17,302 (57.0)	18,015 (59.2)	< 0.001	MDD > HC,BD > SCZ
Ischemic stroke (*n*, %)	173 (0.19)	240 (0.78)	315 (1.04)	326 (1.07)	< 0.001	BD,MDD > SCZ > HC
Age at diagnosis (years, SD)	65.3 (15.2)	62.8 (11.3)	64.2 (12.1)	65.7 (11.3)	0.023	MDD > SCZ
Duration between enrollment and stroke (years, SD)	6.7 (2.6)	4.3 (2.9)	3.8 (2.7)	4.0 (2.8)	< 0.001	HC > SCZ,BD,MDD
Hemorrhagic stroke (*n*, %)	61 (0.07)	90 (0.29)	98 (0.32)	77 (0.25)	< 0.001	SCZ,BD,MDD > HC
Age at diagnosis (years, SD)	60.8 (17.6)	58.5 (14.3)	56.4 (14.7)	53.2 (14.7)	0.032	HC > MDD
Duration between enrollment and stroke (years, SD)	6.6 (2.5)	4.5 (3.1)	4.3 (2.6)	4.2 (3.1)	< 0.001	HC > SCZ,BD,MDD
Acute but ill‐defined cerebrovascular disease (*n*, %)	23 (0.025)	28 (0.090)	37 (0.122)	41 (0.135)	< 0.001	SCZ,BD,MDD > HC
Comorbidities (*n*, %)						
Alcohol use disorder	7453 (8.1)	2642 (8.5)	2569 (8.5)	2242 (7.4)	< 0.001	HC,SCZ,BD > MDD
Hypertension	17,130 (18.7)	6052 (19.6)	5818 (19.2)	5260 (17.3)	< 0.001	HC,SCZ,BD > MDD
Dyslipidemia	12,119 (13.2)	3926 (12.7)	4191 (13.8)	4002 (13.1)	< 0.001	BD > HC,SCZ,MDD
Diabetes mellitus	9495 (10.3)	3325 (10.7)	3268 (10.8)	2902 (9.5)	< 0.001	HC,SCZ,BD > MDD
Traumatic brain injury	3738 (4.1)	1286 (4.2)	1271 (4.2)	1181 (3.9)	< 0.214	
CCI score (SD)	1.02 (1.43)	1.31 (1.62)	1.59 (1.75)	1.71 (1.84)	< 0.001	MDD > BD > SCZ > HC
Smoking (*n*, %)	3400 (3.7)	1439 (4.7)	1392 (4.6)	1261 (4.1)	< 0.001	SCZ,BD > MDD > HC
All‐cause clinical visits (times per year, SD)	6.3 (7.0)	10.3 (12.6)	13.1 (13.8)	13.7 (13.4)	< 0.001	BD > MDD > SCZ > HC
Urbanization level (*n*, %)						
1 (most urbanized)	22,349 (24.4)	7071 (23.0)	7485 (24.8)	7793 (25.8)	< 0.001	
2	30,708 (33.5)	10,230 (33.3)	10,130 (33.6)	10,348 (34.2)		
3	10,565 (11.5)	3684 (12.0)	3401 (11.3)	3480 (11.5)		
4	11,178 (12.2)	3850 (12.5)	3808 (12.6)	3520 (11.6)		
5 (most rural)	16,952 (18.5)	6110 (19.9)	5536 (18.4)	5306 (17.5)		
Income‐related insured amount (*n*, %)^a^						
≤ 15,840 NTD/month	40,049 (43.7)	14,959 (48.7)	12,982 (43.0)	12,108 (40.0)	< 0.001	
15,841–25,000 NTD/month	33,798 (36.9)	11,038 (35.9)	11,285 (37.4)	11,475 (37.9)		
≥ 25,001 NTD/month	17,905 (19.5)	4948 (16.1)	6093 (20.2)	6864 (22.7)		
Antidepressants treatment (*n*, %)						
< 30 cDDD	91,449 (99.7)	20,169 (65.2)	11,836 (39.0)	5955 (19.6)	< 0.001	
30–364 cDDD	288 (0.3)	5342 (17.3)	8535 (28.1)	13,086 (43.0)		
≧365 cDDD	15 (0.0)	5434 (17.6)	9989 (32.9)	11,406 (37.5)		
Lithium treatment (*n*, %)						
< 30 cDDD	91,747 (99.9)	28,541 (92.2)	22,364 (73.7)	30,039 (98.7)	< 0.001	
30–364 cDDD	3 (0.0)	1272 (4.1)	3673 (12.1)	337 (1.1)		
≧365 cDDD	2 (0.0)	1132 (3.7)	4313 (14.2)	71 (0.2)		
Antiepileptic mood stabilizers (*n*, %)						
< 30 cDDD	91,232 (99.4)	24,289 (78.4)	16,266 (53.6)	28,589 (93.9)	< 0.001	
30–364 cDDD	401 (0.4)	4044 (13.1)	8491 (28.0)	1534 (5.0)		
≧365 cDDD	119 (0.1)	2612 (8.4)	5603 (18.5)	324 (1.1)		
Antipsychotics treatment (*n*, %)						
< 30 cDDD	91,685 (99.9)	9284 (30.0)	19,888 (65.5)	28,139 (92.4)	< 0.001	
30–364 cDDD	61 (0.0)	7865 (25.4)	6675 (22.0)	1819 (6.0)		
≧365 cDDD	6 (0.0)	13,796 (44.6)	3797 (12.5)	489 (1.6)		

Abbreviations: CCI, Charlson Comorbidity Index; NTD, new Taiwan dollar; SD, standard deviation.

^a^ 1.00 Taiwanese new dollar = US$0.035.

**FIGURE 2:**
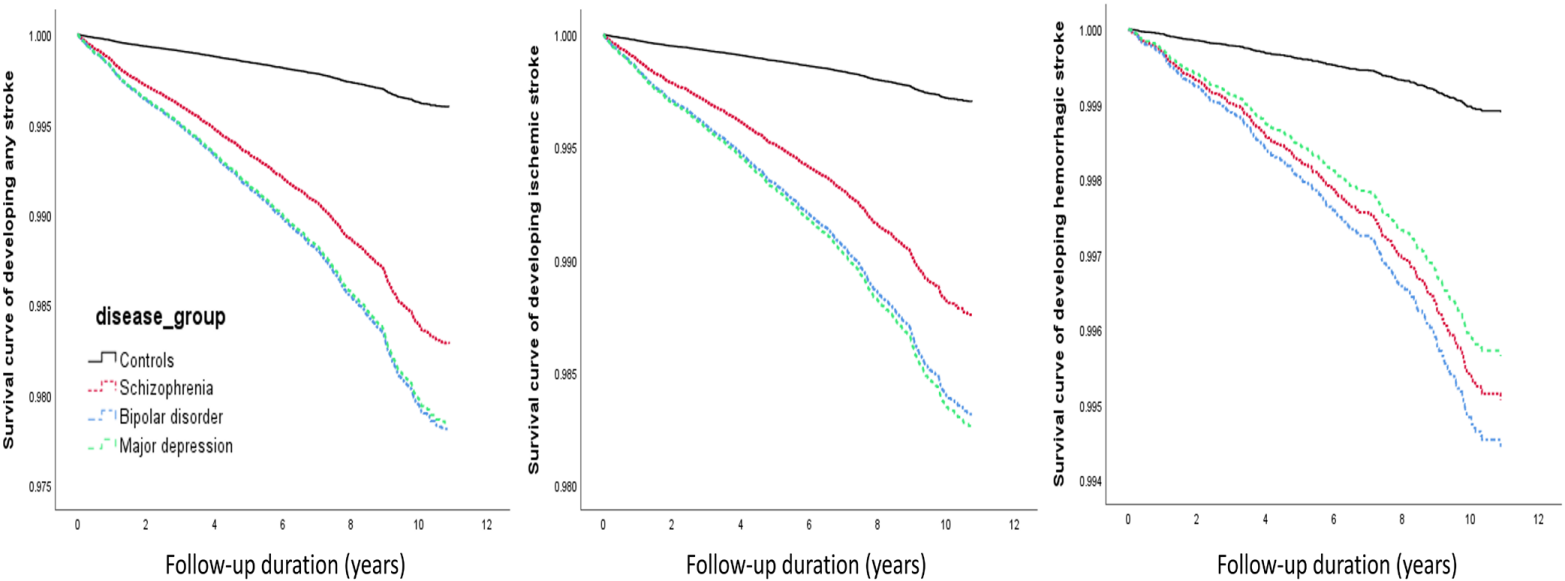
Survival curve of stroke among patients with schizophrenia, bipolar disorder, major depressive disorder, and control group. All *p* < 0.001.

Kaplan–Meier survival analysis confirmed that patients with schizophrenia, bipolar disorder, and major depressive disorder had a significantly elevated risk for any stroke, ischemic stroke, and hemorrhagic stroke compared with the control group (all *p* < 0.001; Figure 2). In Cox regression models adjusted for demographic data, CCI score, smoking, and medical comorbidities, all psychiatric groups were associated with significantly increased risks of developing any stroke, ischemic stroke, and hemorrhagic stroke compared to the control group (Table 2). The risk of developing stroke was especially elevated after additionally adjusting for psychotropic medication use (Table 2). Furthermore, we compared the stroke risk between patients of different psychiatric cohorts with the adjustment of all covariates, but found no differences in stroke risk between psychiatric cohorts.

**TABLE 2:** Risk of developing stroke among patients.

	Model 1		Model 2		Model 3		Model 4	
	Crude HR	95% CI	Adjusted HR	95% CI	Adjusted HR	95% CI	Adjusted HR	95% CI
Any stroke								
Control	1		1		1		1	
Schizophrenia	4.30	3.63–5.09	3.95	3.33–4.69	3.92	3.31–4.66	6.97	5.80–8.37
Bipolar disorder	5.51	4.68–6.48	4.44	3.76–5.24	4.35	3.68–5.14	7.11	5.88–8.59
Major depression	5.42	4.61–6.38	4.19	3.54–4.95	4.18	3.53–4.94	6.05	5.00–7.32
Ischemic stroke								
Control	1		1		1		1	
Schizophrenia	4.20	3.46–5.11	3.86	3.17–4.71	3.84	3.15–4.68	6.84	5.54–8.46
Bipolar disorder	5.71	4.74–6.87	4.52	3.74–5.46	4.40	3.63–5.32	7.15	5.76–8.88
Major depression	5.89	4.90–7.08	4.40	3.64–5.32	4.27	3.52–5.18	6.12	4.93–7.60
Hemorrhagic stroke								
Control	1		1		1		1	
Schizophrenia	4.46	3.22–6.17	4.02	2.89–5.58	4.03	2.90–5.60	7.02	4.91–10.04
Bipolar disorder	5.03	3.65–6.92	4.28	3.09–5.94	4.22	3.04–5.86	6.98	4.77–10.19
Major depression	3.93	2.81–5.50	3.33	2.36–4.70	3.56	2.51–5.04	5.46	3.69–8.08

*Note*: Model 2 adjusted for demographic data (age, sex, income, level of urbanization, all cause clinical visits). Model 3 adjusted for demographic data, Charlson comorbidity index, smoking, and medical comorbidities. Model 4 adjusted for demographic data, Charlson comorbidity index, smoking, medical comorbidities, and psychotropic medication use.

Subgroup analyses using Cox proportional hazards models stratified by age and sex further supported the robustness of the association between psychiatric disorders and stroke risk (Tables S1–S3). Patients aged ≥ 50 years or < 50 years all exhibited increased risks of any stroke, including ischemic and hemorrhagic subtypes across psychiatric cohorts compared to the controls. Stratification by sex revealed that both male and female patients with schizophrenia, bipolar disorder, or major depressive disorder were at significantly higher risk for stroke than controls, with men demonstrating higher hazard ratios than women in the bipolar disorder and major depressive disorder cohorts. Notably, traumatic brain injury, dyslipidemia, and hypertension were consistently associated with increased stroke risk across all subgroups. Regarding the impact of psychotropic medications on stroke risk, higher cumulative exposure to antidepressants was associated with a reduced risk of stroke across all three psychiatric disorders, while higher cumulative exposure to antipsychotics was associated with a reduced risk in the bipolar and schizophrenia cohorts. Lithium did not show significant associations with stroke risk in either bipolar disorder or major depression. Non‐lithium antiepileptic mood stabilizers showed no clear effect in schizophrenia, whereas high cumulative exposure was associated with a protective effect in bipolar disorder.

Mediation analyses using hypertension and dyslipidemia as potential mediators showed minimal and mostly non‐significant indirect effects, with proportions mediated generally close to zero across psychiatric disorders and stroke subtypes (Table S4). These findings suggest that the increased stroke risk associated with psychiatric disorders is primarily attributable to the disorders themselves and is largely independent of mediation by hypertension or dyslipidemia.

In sensitivity analyses, patients with schizophrenia, bipolar disorder, and major depression had an elevated risk of any stroke, ischemic stroke, and hemorrhagic stroke after excluding the first year or the first 3 years of the observation period (Table 3).

**TABLE 3:** Sensitivity test for the risk of developing stroke among patients, adjusted for demographic data (age, sex, income, level of urbanization, all‐cause clinical visits), Charlson comorbidity index, smoking, medical comorbidities, and psychotropic medication use.

	All		> 1 year		> 3 years	
	Adjusted HR	95% CI	Adjusted HR	95% CI	Adjusted HR	95% CI
Any stroke						
Control	1		1		1	
Schizophrenia	6.97	5.80–8.37	5.56	4.58–6.77	4.07	3.26–5.08
Bipolar disorder	7.11	5.88–8.59	5.19	4.23–6.37	3.63	2.87–4.59
Major depression	6.05	5.00–7.32	4.57	3.72–5.60	3.07	2.42–3.89
Ischemic stroke						
Control	1		1		1	
Schizophrenia	6.84	5.54–8.46	5.48	4.37–6.88	4.10	3.17–5.31
Bipolar disorder	7.15	5.76–8.88	5.05	3.99–6.39	3.41	2.60–4.48
Major depression	6.12	4.93–7.60	4.57	3.62–5.77	3.11	2.37–4.08
Hemorrhagic stroke						
Control	1		1		1	
Schizophrenia	7.02	4.91–10.04	5.59	3.83–8.16	3.97	2.56–6.13
Bipolar disorder	6.98	4.77–10.19	5.59	3.74–8.35	4.53	2.88–7.12
Major depression	5.46	3.69–8.08	4.18	2.75–6.36	2.70	1.65–4.42

## 4 Discussion

In this large‐scale, population‐based cohort study using Taiwan's NHIRD, we found that individuals with schizophrenia, bipolar disorder, or major depressive disorder had significantly higher risks of incident stroke, including ischemic and hemorrhagic subtypes, compared with age‐matched controls without psychiatric illness. These findings align with prior evidence suggesting that psychiatric disorders are associated with heightened vascular risk; however, our study extends this literature by offering a direct comparison across multiple psychiatric diagnoses within a unified cohort and analytical framework.

The increased stroke risk remained robust across diverse demographic strata. Subgroup analyses demonstrated elevated stroke risk in both younger and older adults and in both sexes, supporting the generalizability of these findings across age and gender. These results underscore the systemic nature of cerebrovascular vulnerability in psychiatric populations, beyond the influence of specific demographic or clinical profiles. This finding is clinically important, as younger patients with psychiatric disorders are often not recognized as being at elevated vascular risk, yet our data suggest they warrant targeted stroke prevention strategies [23]. Furthermore, stroke risk remained consistently elevated regardless of sex, although slightly higher hazard ratios were observed in males across diagnostic groups, a trend that aligns with population‐based stroke risk models [24].

Regarding the role of psychotropic medications in stroke risk among patients with major psychiatric disorders, our findings are partly consistent with prior literature but also highlight several important divergences. Previous case‐crossover research reported an elevated risk of stroke among patients with schizophrenia within a short exposure window of second‐generation antipsychotics, particularly with agents such as quetiapine and zotepine, and suggested a receptor affinity‐related acute effect [14, 15]. In contrast, our nationwide cohort demonstrated that long‐term cumulative exposure to second‐generation antipsychotics in patients with schizophrenia was associated with a reduced rather than elevated risk of stroke. Several methodological distinctions may account for these differences. The case‐crossover design is optimized for assessing transient exposures and acute outcomes, whereas our longitudinal cohort approach captured cumulative treatment patterns over years of follow‐up. Moreover, our analysis incorporated a population‐based control cohort and adjusted for a wide range of comorbidities and socioeconomic factors, underscoring the importance of study design and time horizon when evaluating cerebrovascular safety. These findings are also in line with reports from other psychiatric populations suggesting that prolonged antipsychotic use may not uniformly elevate stroke risk; for example, Hsieh et al. found no significant association between second‐generation antipsychotics and cerebrovascular events in a cohort of 9715 newly diagnosed patients with schizophrenia [25]. Similarly, our results in patients with bipolar disorder differ from those of a prior nested case–control study using the same NHIRD database, which reported that second‐generation antipsychotic use was associated with increased stroke risk [26]. Again, differences in study design and exposure definition may account for these discrepancies. The nested case–control study evaluated drug use during the 6 months preceding a stroke event, emphasizing the presence versus absence of drug exposure, whereas our approach incorporated dose–response relationships based on cumulative defined daily doses over years of follow‐up. Our design allowed us to detect potential protective associations with long‐term exposure to second‐generation antipsychotics, as sustained treatment may stabilize symptoms in schizophrenia and bipolar disorder, improving care continuity and mitigating vascular risk despite possible short‐term adverse effects [27, 28].

Across schizophrenia, bipolar disorder, and major depression, we found that long‐term antidepressant exposure was associated with a lower risk of stroke. This differs from some meta‐analyses in mixed or general populations that report modest increases in stroke, especially with selective serotonin reuptake inhibitors (SSRIs), including a pooled 41% higher risk [29]. By contrast, prospective data limited to non‐cardioembolic ischemic stroke and studies of middle‐aged adults with depression show no significant association between SSRIs and stroke [18, 30]. Consistent with our findings, a nationwide obsessive‐compulsive disorder cohort observed lower incident stroke with ≥ 2 months of SSRI use; the authors proposed a time‐dependent pattern of acute detriment but chronic benefit in reducing thrombotic events via platelet serotonin depletion and reduced aggregation [31]. Taken together, these observations suggest that time window and patient selection matter: risk may rise transiently around initiation, whereas longer‐term, adherent treatment during clinical stability may align with lower stroke incidence [32, 33]. Still, our findings should be interpreted cautiously because common patterns in observational data can make long‐term users look safer than they truly are. For example, patients who persist with treatment often also engage in healthier behaviors. Future studies should separate risks around treatment initiation versus maintenance periods, and track medication use as it changes over time while comparing patients who newly start antidepressants with similar patients who newly start another indicated therapy. These approaches better address confounding and improve how well the findings generalize across psychiatric populations.

Our finding that lithium was not significantly associated with stroke risk in bipolar disorder contrasts with several prior observational studies suggesting a potential protective effect [16]. By contrast, Chen et al. demonstrated that acute lithium exposure was not associated with stroke risk, and our results are concordant with this observation [17]. Overall, using a nationwide database with a large sample size, our results support the notion that lithium does not increase stroke risk in major psychiatric populations. Notably, the observed increase in hazard ratios after adjusting for psychotropic medication use in Table 2 should not be interpreted as evidence that these treatments heighten stroke risk. Psychotropic exposure likely functions as a mediator in the pathway linking psychiatric disorders to stroke, and may also serve as a proxy for illness severity and health care engagement. If such treatments exert partial protective effects, for example, by improving lifestyle stability or facilitating access to medical care, statistical adjustment may truncate this pathway and yield a suppression effect, making the independent contribution of psychiatric disorders appear more pronounced [34].

The sensitivity analyses in this study provide important additional insights. By excluding the first 1 or 3 years of follow‐up, these models aim to mitigate potential biases from reverse causality, that is, the possibility that incipient cerebrovascular disease may influence the onset or exacerbation of psychiatric illness, rather than psychiatric disorders predisposing to stroke. The persistence of significantly elevated risks for any stroke, ischemic stroke, and hemorrhagic stroke across schizophrenia, bipolar disorder, and major depressive disorder after the exclusion of early follow‐up periods reinforces the robustness of the observed associations and argues against reverse causality as the sole explanation. These findings suggest that the cerebrovascular risk among individuals with severe psychiatric disorders is sustained over time and is unlikely to be entirely attributable to short‐term clustering of events around the time of psychiatric diagnosis. Instead, long‐term pathophysiological mechanisms, including chronic systemic inflammation, metabolic dysregulation, hypothalamic–pituitary–adrenal axis abnormalities, and adverse lifestyle factors, may contribute to enduring vascular vulnerability. At the same time, the relatively low incidence of hemorrhagic stroke warrants cautious interpretation, and replication in larger pooled cohorts will be essential to confirm these results. Collectively, our analyses underscore the need for continuous cardiovascular surveillance and prevention strategies throughout the course of psychiatric illness, rather than only in its early stages.

These findings reinforce the growing recognition that psychiatric illness constitutes an independent and clinically relevant risk factor for cerebrovascular disease, extending prior research that typically examined psychiatric diagnoses in isolation and rarely accounted for stroke subtypes. Our study underscored clinical implications, particularly the need for incorporating cerebrovascular risk assessment and prevention into psychiatric care. Most existing cardiovascular or stroke risk prediction models, such as the Framingham Stroke Risk Profile [35], do not account for psychiatric diagnoses. Given the persistently elevated risk of stroke observed across psychiatric groups, even after adjusting for conventional vascular comorbidities, psychiatric illness may function as a distinct, independent risk modifier. Therefore, integrating severe mental illness into existing risk stratification tools may improve the identification of high‐risk individuals, especially among younger patients who might otherwise fall outside traditional age‐based criteria. Future work should examine whether stroke risk can be mitigated through psychiatric treatment optimization.

This study has several limitations. First, although the use of the NHIRD allowed for comprehensive, population‐based analyses with high generalizability, its reliance on administrative claims data may have introduced diagnostic misclassification. Nonetheless, we employed validated coding strategies and applied stringent criteria (e.g., diagnosis by board‐certified physicians, multiple claims, principal inpatient diagnoses) to enhance specificity. Second, we performed separate 1:1 matching for each psychiatric disorder group and subsequently merged the matched controls into a single pooled control cohort, an approach that has been adopted in previous NHIRD‐based studies [19, 36]. To ensure minimal heterogeneity, we assessed the baseline covariates across all groups, and found that the maximum absolute standardized difference was < 0.1, suggesting a balanced cohort. Nevertheless, the possibility of residual heterogeneity from merging matched controls warrants caution in interpreting between‐group comparisons. Furthermore, despite adjusting for a wide range of covariates, our study may still be subject to residual confounding, particularly from unmeasured lifestyle factors such as physical activity, dietary habits, and medication adherence. The use of an insurance claims database further limits access to potentially important confounding variables, including family history, severity of psychiatric illness, and environmental exposures. Third, our smoking definition, based on cessation pharmacotherapy and ICD‐9‐CM codes, may have led to misclassification by capturing primarily treatment‐seeking individuals while missing untreated or uncoded smokers, potentially underestimating prevalence and introducing residual bias. Finally, information on stroke severity, recurrence, or post‐stroke outcomes was not available, which limits the assessment of long‐term prognosis.

In conclusion, this nationwide cohort study demonstrates that schizophrenia, bipolar disorder, and major depressive disorder are independently associated with increased risks of ischemic and hemorrhagic stroke compared to the general population. The risk is robust across age and sex strata and persists over time. These findings emphasize the need for integrated care, stroke risk surveillance, and future mechanistic research tailored to psychiatric populations.

## Author Contributions

**Mao‐Hsuan Huang:** formal analysis, investigation, methodology, writing – original draft preparation. **Chih‐Ming Cheng:** formal analysis, methodology. **Ju‐Wei Hsu:** supervision, validation. **Ya‐Mei Bai:** funding acquisition. **Tung‐Ping Su:** supervision, validation. **Cheng‐Ta Li:** funding acquisition. **Shih‐Jen Tsai:** funding acquisition. **Yee‐Lam E. Chan:** validation. **Mu‐Hong Chen:** conceptualization, data curation, formal analysis, funding acquisition, investigation, methodology, project administration, resources, writing – review and editing.

## Conflicts of Interest

The authors declare no conflicts of interest.

## Supporting information


**Table S1:** Cox regression analyses of the risk of any stroke among patients with schizophrenia and controls, adjusted for demographic data (age, sex, income, level of urbanization, all cause clinical visits), Charlson comorbidity index (CCI), smoking, and medical comorbidities.
**Table S2:** Cox regression analyses of the risk of stroke among patients with bipolar disorder and controls, adjusted for demographic data (age, sex, income, level of urbanization, all cause clinical visits), Charlson comorbidity index (CCI), smoking, and medical comorbidities.
**Table S3:** Cox regression analyses of the risk of stroke among patients with major depression and controls, adjusted for demographic data (age, sex, income, level of urbanization, all cause clinical visits), Charlson comorbidity index (CCI), smoking, and medical comorbidities.
**Table S4:** Natural direct, indirect, and total effects of hypertension or dyslipidemia on the occurrence of stroke among patients with psychiatric disorders.

## Data Availability

The data that support the findings of this study are available on request from the corresponding author. The data are not publicly available due to privacy or ethical restrictions.
