# Supplementary material for: Risk of Stroke in Patients With Schizophrenia, Bipolar Disorder, and Major Depressive Disorder: A Cohort Study of 183,504 Subjects
Source: Acta Psychiatr Scand. 2025 Oct 30;153(1):54–64. doi: 10.1111/acps.70043 (PMC12668889; doi:10.1111/acps.70043)
Supplement: Supplementary file 1 — Table S1: Cox regression analyses of the risk of any stroke among patients with schizophrenia and controls, adjusted for demographic data (age, sex, income, level of urbanization, all cause clinical visits), Charlson comorbidity index (CCI), smoking, and medical comorbidities. Table S2: Cox regression analyses of the risk of stroke among patients with bipolar disorder and controls, adjusted for demographic data (age, sex, income, level of urbanization, all cause clinical visits), Charlson comorbidity index (CCI), smoking, and medical comorbidities. Table S3: Cox regression analyses of the risk of stroke among patients with major depression and controls, adjusted for demographic data (age, sex, income, level of urbanization, all cause clinical visits), Charlson comorbidity index (CCI), smoking, and medical comorbidities. Table S4: Natural direct, indirect, and total effects of hypertension or dyslipidemia on the occurrence of stroke among patients with psychiatric disorders. [file ACPS-153-54-s001.docx]

Supplementary Table 1 Cox regression analyses of the risk of any stroke among patients with schizophrenia and controls, adjusted for demographic data (age, sex, income, level of urbanization, all cause clinical visits), Charlson comorbidity index (CCI), smoking, and medical comorbidities

|  | Age <50 y  HR (95% CI) | Age ≥50 y  HR (95% CI) | Males  HR (95% CI) | Females  HR (95% CI) | Total  HR (95% CI) |
| --- | --- | --- | --- | --- | --- |
| Schizophrenia  (presence v. absence) | **4.05 (2.69-6.11)** | **8.02 (6.32-10.18)** | **4.73 (3.43-6.51)** | **8.89 (6.78-11.65)** | **6.83 (5.57-8.39)** |
| Age at enrollment | **1.05 (1.02-1.07)** | **1.02 (1.01-1.04)** | **1.02 (1.01-1.03)** | **1.04 (1.03-1.05)** | **1.03 (1.03-1.04)** |
| Male | **1.55 (1.11-2.17)** | 0.93 (0.75-1.15) |  |  | 1.05 (0.88-1.25) |
| All-cause clinical visits | 1.01 (1.00-1.02) | 1.00 (0.99-1.01) | 1.00 (1.00-1.01) | 1.00 (1.00-1.01) | 1.00 (1.00-1.01) |
| CCI score | **1.34 (1.24-1.44)** | **1.20 (1.15-1.25)** | **1.21 (1.15-1.28)** | **1.23 (1.17-1.29)** | **1.22 (1.17-1.26)** |
| Comorbidities |  |  |  |  |  |
| Alcohol use | 1.41 (0.93-2.16) | 1.00 (0.61-1.65) | 1.36 (0.96-1.93) | 1.10 (0.52-2.33) | **1.38 (1.01-1.88)** |
| Hypertension | **3.75 (2.58-5.46)** | **3.76 (2.85-4.95)** | **4.78 (3.38-6.74)** | **3.59 (2.66-4.85)** | **4.08 (3.25-5.11)** |
| Dyslipidemia | **1.65 (1.13-2.41)** | 1.25 (1.00-1.57) | **1.47 (1.08-1.98)** | **1.38 (1.07-1.77)** | **1.43 (1.18-1.73)** |
| Diabetes mellitus | 1.06 (0.70-1.61) | 0.96 (0.76-1.22) | 1.08 (0.79-1.48) | 0.99 (0.76-1.29) | 1.01 (0.82-1.23) |
| Traumatic brain injury | **3.81 (2.24-6.47)** | **1.75 (1.19-2.56)** | **2.57 (1.61-4.10)** | **1.95 (1.29-2.94)** | **2.14 (1.57-2.92)** |
| Antidepressants treatment |  |  |  |  |  |
| <30 cDDD | 1 | 1 | 1 | 1 | 1 |
| 30–364 cDDD | 0.83 (0.47-1.47) | **0.50 (0.32-0.80)** | 0.84 (0.50-1.42) | **0.47 (0.29-0.72)** | **0.60 (0.42-0.84)** |
| ≧365 cDDD | **0.42 (0.21-0.85)** | 0.71 (0.47-1.08) | 0.69 (0.39-1.21) | **0.57 (0.36-0.90)** | **0.61 (0.43-0.88)** |
| Antipsychotics treatment |  |  |  |  |  |
| <30 cDDD | 1 | 1 | 1 | 1 | 1 |
| 30–364 cDDD | 0.92 (0.55-1.54) | **0.68 (0.50-0.92)** | 0.87 (0.57-1.32) | **0.61 (0.44-0.85)** | **0.73 (0.56-0.94)** |
| ≧365 cDDD | **0.34 (0.20-0.60)** | **0.31 (0.22-0.44)** | **0.32 (0.20-0.53)** | **0.30 (0.20-0.43)** | **0.32 (0.24-0.43)** |
| Lithium treatment |  |  |  |  |  |
| <30 cDDD | 1 | 1 | 1 | 1 | 1 |
| 30–364 cDDD | 1.06 (0.33-3.46) | 1.42 (0.62-3.27) | 1.44 (0.52-4.00) | 1.10 (0.44-2.73) | 1.24 (0.63-2.44) |
| ≧365 cDDD | **2.64 (1.19-5.85)** | 1.82 (0.83-3.98) | **2.54 (1.12-5.78)** | 1.86 (0.87-3.97) | **2.10 (1.21-3.66)** |
| Antiepileptic mood stabilizers |  |  |  |  |  |
| <30 cDDD | 1 | 1 | 1 | 1 | 1 |
| 30–364 cDDD | 1.29 (0.72-2.32) | 0.98 (0.64-1.50) | 0.90 (0.53-1.51) | 1.23 (0.78-1.95) | 1.02 (0.72-1.43) |
| ≧365 cDDD | 1.04 (0.51-2.08) | 0.44 (0.18-1.09) | 0.72 (0.35-1.50) | 0.72 (0.33-1.59) | 0.72 (0.42-1.23) |

Bold font indicates statistical significance.

Supplementary Table 2 Cox regression analyses of the risk of stroke among patients with bipolar disorder and controls, adjusted for demographic data (age, sex, income, level of urbanization, all cause clinical visits), Charlson comorbidity index (CCI), smoking, and medical comorbidities

|  | Age <50 y  HR (95% CI) | Age ≥50 y  HR (95% CI) | Males  HR (95% CI) | Females  HR (95% CI) | Total  HR (95% CI) |
| --- | --- | --- | --- | --- | --- |
| Bipolar disorder  (presence v. absence) | **4.32 (2.75-6.78)** | **8.78 (6.81-11.31)** | **7.59 (5.59-10.32)** | **6.94 (5.06-9.51)** | **7.30 (5.87-9.08)** |
| Age at enrollment | **1.03 (1.00-1.05)** | **1.03 (1.02-1.04)** | **1.03 (1.02-1.04)** | **1.05 (1.04-1.06)** | **1.04 (1.03-1.04)** |
| Male | **1.61 (1.17-2.22)** | 1.18 (0.97-1.43) |  |  | **1.29 (1.10-1.52)** |
| All-cause clinical visits | 1.01 (0.99-1.02) | **1.01 (1.00-1.01)** | 1.00 (0.99-1.01) | **1.01 (1.01-1.02)** | **1.01 (1.00-1.01)** |
| CCI score | **1.33 (1.24-1.43)** | **1.21 (1.16-1.26)** | **1.22 (1.16-1.29)** | **1.25 (1.19-1.32)** | **1.23 (1.19-1.28)** |
| Comorbidities |  |  |  |  |  |
| Alcohol use | 1.37 (0.90-2.08) | 0.63 (0.35-1.13) | 0.89 (0.61-1.30) | 1.92 (1.04-3.53) | 1.11 (0.80-1.53) |
| Hypertension | **4.27 (2.98-6.12)** | **2.49 (1.97-3.15)** | **3.98 (2.97-5.32)** | **2.32 (1.74-3.09)** | **3.13 (2.55-3.83)** |
| Dyslipidemia | **1.48 (1.02-2.14)** | 1.01 (0.82-1.25) | 1.16 (0.89-1.52) | 1.15 (0.90-1.48) | 1.16 (0.97-1.39) |
| Diabetes mellitus | 1.20 (0.80-1.79) | 1.06 (0.86-1.31) | 1.17 (0.90-1.53) | 1.04 (0.80-1.35) | 1.11 (0.92-1.33) |
| Traumatic brain injury | **4.32 (2.75-6.78)** | **2.50 (1.83-3.42)** | 2.59 (1.78-3.77) | **3.07 (2.16-4.37)** | **2.79 (2.16-3.60)** |
| Antidepressants treatment |  |  |  |  |  |
| <30 cDDD | 1 | 1 | 1 | 1 | 1 |
| 30–364 cDDD | 0.84 (0.50-1.40) | **0.59 (0.44-0.78)** | **0.62 (0.43-0.89)** | **0.65 (0.45-0.92)** | **0.62 (0.49-0.80)** |
| ≧365 cDDD | 0.85 (0.53-1.35) | **0.52 (0.40-0.68)** | **0.65 (0.46-0.90)** | **0.55 (0.40-0.76)** | **0.59 (0.47-0.75)** |
| Antipsychotics treatment |  |  |  |  |  |
| <30 cDDD | 1 | 1 | 1 | 1 | 1 |
| 30–364 cDDD | 0.97 (0.58-1.62) | **0.58 (0.42-0.80)** | 0.77 (0.52-1.12) | **0.58 (0.39-0.85)** | **0.68 (0.52-0.90)** |
| ≧365 cDDD | **0.24 (0.09-0.69)** | **0.30 (0.17-0.54)** | **0.14 (0.50-0.40)** | **0.39 (0.22-0.72)** | **0.29 (0.17-0.48)** |
| Lithium treatment |  |  |  |  |  |
| <30 cDDD | 1 | 1 | 1 | 1 | 1 |
| 30–364 cDDD | 0.99 (0.55-1.76) | **1.69 (1.19-2.41)** | 1.43 (0.94-2.17) | 1.43 (0.92-2.21) | 1.44 (1.06-1.94) |
| ≧365 cDDD | 0.77 (0.43-1.39) | 0.81 (0.54-1.23) | 0.62 (0.37-1.04) | 1.07 (0.69-1.67) | 0.81 (0.58-1.13) |
| Antiepileptic mood stabilizers |  |  |  |  |  |
| <30 cDDD | 1 | 1 | 1 | 1 | 1 |
| 30–364 cDDD | 1.14 (0.72-1.78) | 1.09 (0.83-1.43) | 1.06 (0.76-1.46) | 1.13 (0.81-1.57) | 1.08 (0.86-1.36) |
| ≧365 cDDD | 0.71 (0.40-1.27) | **0.46 (0.29-0.74)** | **0.58 (0.33-0.93)** | **0.55 (0.33-0.93)** | **0.53 (0.37-0.77)** |

Bold font indicates statistical significance.

Supplementary Table 3 Cox regression analyses of the risk of stroke among patients with major depression and controls, adjusted for demographic data (age, sex, income, level of urbanization, all cause clinical visits), Charlson comorbidity index (CCI), smoking, and medical comorbidities

|  | Age <50 y  HR (95% CI) | Age ≥50 y  HR (95% CI) | Males  HR (95% CI) | Females  HR (95% CI) | Total  HR (95% CI) |
| --- | --- | --- | --- | --- | --- |
| Major depression  (presence v. absence) | **4.25 (2.59-6.97)** | **6.58 (4.98-8.68)** | **6.54 (4.73-9.05)** | **5.06 (3.53-7.25)** | **5.75 (4.53-7.31)** |
| Age at enrollment | **1.04 (1.02-1.07)** | **1.04 (1.03-1.05)** | **1.03 (1.03-1.04)** | **1.05 (1.04-1.06)** | **1.04 (1.03-1.05)** |
| Male | **1.65 (1.19-2.30)** | **1.36 (1.13-1.65)** |  |  | **1.42 (1.21-1.68)** |
| All-cause clinical visits | 1.01 (1.00-1.03) | **1.01 (1.00-1.01)** | **1.01 (1.00-1.02)** | 1.01 (0.99-1.01) | **1.01 (1.00-1.01)** |
| CCI score | **1.30 (1.21-1.40)** | **1.19 (1.14-1.24)** | **1.19 (1.13-1.25)** | **1.24 (1.18-1.30)** | **1.21 (1.17-1.26)** |
| Comorbidities |  |  |  |  |  |
| Alcohol use | 1.48 (0.98-2.22) | 1.05 (0.66-1.66) | 1.30 (0.92-1.83) | 1.78 (0.97-3.27) | 1.41 (0.95-1.90) |
| Hypertension | **4.46 (3.08-6.46)** | **2.54 (2.01-3.20)** | **3.35 (2.53-4.45)** | **2.98 (2.21-4.03)** | **3.17 (2.59-3.90)** |
| Dyslipidemia | 1.05 (0.71-1.56) | 1.20 (0.98-1.48) | 1.19 (0.92-1.55) | 1.28 (1.00-1.65) | **1.25 (1.05-1.50)** |
| Diabetes mellitus | 1.46 (0.96-2.21) | 0.96 (0.78-1.19) | 0.96 (0.74-1.26) | 1.12 (0.86-1.46) | 1.04 (0.86-1.25) |
| Traumatic brain injury | **5.91 (3.79-9.22)** | **1.61 (1.09-2.37)** | **2.47 (1.65-3.69)** | **2.24 (1.48-3.38)** | **2.34 (1.76-3.12)** |
| Antidepressants treatment |  |  |  |  |  |
| <30 cDDD | 1 | 1 | 1 | 1 | 1 |
| 30–364 cDDD | 0.87 (0.51-1.47) | 0.93 (0.71-1.22) | 0.85 (0.61-1.18) | 1.01 (0.70-1.45) | 0.92 (0.72-1.18) |
| ≧365 cDDD | **0.50 (0.28-0.90)** | **0.49 (0.37-0.67)** | **0.49 (0.34-0.70)** | **0.54 (0.37-0.80)** | **0.51 (0.39-0.67)** |
| Antipsychotics treatment |  |  |  |  |  |
| <30 cDDD | 1 | 1 | 1 | 1 | 1 |
| 30–364 cDDD | 0.32 (0.08-1.35) | **0.41 (0.23-0.74)** | 0.56 (0.30-1.08) | **0.22 (0.08-0.60)** | **0.39 (0.23-0.67)** |
| ≧365 cDDD | 0.59 (0.08-4.38) | 0.46 (0.17-1.24) | 0.28 (0.04-2.03) | 0.51 (0.18-1.41) | 0.45 (0.19-1.11) |
| Lithium treatment |  |  |  |  |  |
| <30 cDDD | 1 | 1 | 1 | 1 | 1 |
| 30–364 cDDD | 1.00 (0.14-7.26) | 1.48 (0.37-5.99) | 2.26 (0.55-9.21) | 0.78 (0.11-5.57) | 1.34 (0.43-4.21) |
| ≧365 cDDD | 0 (0-0) | **5.22 (1.27-21.46)** | 3.03 (0.42-21.86) | 3.08 (0.40-23.55) | 3.37 (0.83-13.72) |
| Antiepileptic mood stabilizers |  |  |  |  |  |
| <30 cDDD | 1 | 1 | 1 | 1 | 1 |
| 30–364 cDDD | **2.20 (1.23-3.95)** | 0.76 (0.47-1.24) | 1.03 (0.62-1.72) | 1.0 (0.59-1.71) | 1.03 (0.71-1.48) |
| ≧365 cDDD | 0.64 (0.09-4.71) | 0.57 (0.18-1.83) | 0.30 (0.04-2.18) | 1.14 (0.35-3.70) | 0.68 (0.25-1.85) |

Bold font indicates statistical significance.

Supplementary Table 4 Natural direct, indirect, and total effects of hypertension or dyslipidemia on the occurrence of stroke among patients with psychiatric disorders

| Psychiatric diagnosis | Stroke type | mediator | Natural indirect effect (HR, 95%CI) | Natural direct effect (HR, 95%CI) | Natural total effect (HR, 95%CI) | Proportion mediated, % |
| --- | --- | --- | --- | --- | --- | --- |
| schizophrenia | Ischemic | Hypertension | 1.00 (0.99-1.02) | 6.96 (4.88-9.03) | 6.97 (4.89-9.05) | 0.25 |
|  | Ischemia | dyslipidemia | 0.97 (0.96-0.99) | 6.63 (4.96-8.29) | 6.45 (4.81-8.10) | -3.18 |
|  | Hemorrhagic | hypertension | 1.00 (0.98-1.03) | 7.36 (4.22-10.50) | 7.36 (2.63-12.08) | 0.35 |
|  | Hemorrhagic | dyslipidemia | 1.00 (0.99-1.01) | 6.67 (4.00-9.33) | 6.6 (3.98-9.34) | -0.05 |
| Bipolar disorder | Ischemic | Hypertension | 1.00 (0.99-1.01) | 8.02 (5.81-10.23) | 8.04 (5.82-10.25) | 0.19 |
|  | Ischemia | dyslipidemia | 0.99 (0.98-1.00) | 7.89 (5.81-9.98) | 7.83 (5.75-9.91) | -0.97 |
|  | Hemorrhagic | hypertension | 1.00 (0.99-1.01) | 5.37 (3.00-7.73) | 5.38 (3.01-7.75) | 0.26 |
|  | Hemorrhagic | dyslipidemia | 1.00 (1.00-1.02) | 5.52 (2.95-8.08) | 5.56 (2.97-8.15) | 1.00 |
| Major depressive disorder | Ischemic | Hypertension | 0.97 (0.96-0.98) | 6.59 (4.89-8.30) | 6.42 (4.74-8.10) | -3.18 |
|  | Ischemia | dyslipidemia | 1.00 (1.00-1.01) | 6.46 (5.11-7.82) | 6.48 (5.12-7.84) | 0.27 |
|  | Hemorrhagic | hypertension | 0.97 (0.95-1.00) | 7.59 (3.64-11.55) | 7.36 (3.50-11.23) | -3.57 |
|  | Hemorrhagic | dyslipidemia | 1.01 (1.00-1.01) | 6.24 (3.06-9.43) | 6.28 (3.07-9.48) | 0.61 |
